# Efficient second-harmonic imaging of collagen in histological slides using Bessel beam excitation

**DOI:** 10.1038/srep29863

**Published:** 2016-07-20

**Authors:** Nelly Vuillemin, Pierre Mahou, Delphine Débarre, Thierry Gacoin, Pierre-Louis Tharaux, Marie-Claire Schanne-Klein, Willy Supatto, Emmanuel Beaurepaire

**Affiliations:** 1 Laboratory for optics and biosciences, Ecole polytechnique, CNRS, INSERM, Université Paris-Saclay, 91128 Palaiseau cedex, France; 2 Laboratory of interdisciplinary physics, Université Joseph Fourier, CNRS, 38402 St Martin d’Hères, France; 3 Laboratory of condensed matter physics, Ecole polytechnique, CNRS, Université Paris-Saclay, 91128 Palaiseau cedex, France; 4 Paris-Cardiovascular Research Centre, INSERM, European Georges Pompidou Hospital, 75015 Paris, France

## Abstract

Second-harmonic generation (SHG) is the most specific label-free indicator of collagen accumulation in widespread pathologies such as fibrosis, and SHG-based measurements hold important potential for biomedical analyses. However, efficient collagen SHG scoring in histological slides is hampered by the limited depth-of-field of usual nonlinear microscopes relying on focused Gaussian beam excitation. In this work we analyze theoretically and experimentally the use of Bessel beam excitation to address this issue. Focused Bessel beams can provide an axially extended excitation volume for nonlinear microscopy while preserving lateral resolution. We show that shaping the focal volume has consequences on signal level and scattering directionality in the case of coherent signals (such as SHG) which significantly differ from the case of incoherent signals (two-photon excited fluorescence, 2PEF). We demonstrate extended-depth SHG-2PEF imaging of fibrotic mouse kidney histological slides. Finally, we show that Bessel beam excitation combined with spatial filtering of the harmonic light in wave vector space can be used to probe collagen accumulation more efficiently than the usual Gaussian excitation scheme. These results open the way to SHG-based histological diagnoses.

Collagen is the most abundant protein in the extracellular matrix, and plays a central role in the formation of fibrillar structures in connective tissues^1^. Collagen molecules exhibit a triple helical structure and, in most collagen types (fibrillar collagens), self-assemble into fibrils 10–300 nm in diameter and up to several hundreds of μm in length. Collagen fibrils are found in a wide variety of tissues such as bone, tendon, skin, ligament, cornea and internal organs. Fibrils further assemble into higher order tissue-specific structures, such as bundles or fibers. Proper macromolecular organization of collagens is crucial for the organs’ architecture and function. However, extracellular matrix remodeling may occur in tissues in response to various injuries, and in most cases, it exhibits abnormal accumulation of fibrillar collagen. This process is called fibrosis and eventually leads to organ functional failure. Implementation of reliable fibrosis indexes is currently much needed to better understand the mechanisms regulating collagen remodeling. In the past decade, second harmonic generation (SHG) microscopy has emerged as a promising direction to implement fibrosis assays, since fibrillar collagens intrinsically exhibit strong SHG signals^2^,^3^,^4^,^5^. SHG microscopy is a three-dimensional (3D)-resolved laser scanning technique where a tightly focused Gaussian beam is scanned across the sample while SHG signals are recorded. At the molecular scale, the second harmonic response of collagen originates from the nonlinear polarization of non-centrosymmetric peptide bonds along the collagen triple helix. At the scale of the diffraction-limited probe volume, the signal scales as the square number of aligned peptide bonds within the micron-sized scanned probe volume^6^,^7^,^8^. Collagen SHG is therefore label-free, specific and sensitive, which makes it a good candidate for systematic studies based on fibrosis scoring^9^,^10^,^11^ in histological sections with better reliability than conventional staining-based techniques.

However the generalization of this approach is hampered by the fact that histological sections generally exhibit inhomogeneous thickness and poor flatness, so that SHG-based probing with a point-scanning system requires time-consuming 3D scans. In addition, it is difficult to derive a quantitative interpretation of 3D SHG image stacks in terms of collagen density, since the contrast mechanism is coherent while the scanning process is not. One workaround could be to extend the depth-of-field of the microscope by using a weakly focused excitation Gaussian beam to remove the need for an axial scanning^12^, but this solution necessarily limits the lateral resolution of the image and reduces its information content. There is a need for images revealing both the fibrosis microstructure and its extent over scales of several millimeters. In this context, Bessel beam excitation, recently proposed for multiphoton microscopy applications^13^,^14^,^15^,^16^, appears as an interesting alternative to Gaussian beam excitation in SHG microscopy of tissue sections. Indeed, a focused Bessel beam can in principle provide a nonlinear probe volume with an axial extension encompassing the thickness and height heterogeneity of a histological section (i.e. several microns or several tens of microns), and a confined lateral extension enabling to probe the sample lateral organization. However the use of Bessel beams for second-harmonic generation imaging has not been precisely described so far.

In this article, we analyze and demonstrate the use of Bessel beam excitation for extended-depth SHG imaging of thick samples. We first describe the production of Bessel beams with adjustable properties in a scanning multiphoton microscope. We then demonstrate extended depth SHG-2PEF imaging of fibrotic mouse kidney biopsy samples. Finally, we analyze more precisely the imaging properties of Bessel beams for SHG and two-photon-excited fluorescence (2PEF) microscopy in terms of signal level and emission directionality as a function of sample geometry. Overall, these results show how Bessel beam excitation can be used to probe collagen accumulation more rapidly and more reliably than the usual Gaussian excitation scheme.

## Results and Discussion

### Extended depth-of-focus nonlinear imaging with Bessel beams

#### Bessel beams

Bessel beams are a class of beams that were first introduced by Durnin *et al*. in 1987^17^,^18^,^19^ and that exhibit an invariant intensity distribution along their propagation axis, producing a “needle” of light of infinite extension. Such an ideal Bessel beam is defined by the following equation:
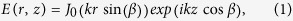
where *J*_*0*_ is the 0-order Bessel function of the first kind, r is the distance from the optical axis in a transverse plane, z is the propagation distance along the optical axis, k is the wave vector and *β* the angle between the optical axis and the wave vectors forming the Bessel beam (see Supplementary figure S1). A Bessel beam is obtained when focusing an infinitely thin ring. However it should be noted that, similarly to plane waves, ideal Bessel beams carry an infinite amount of energy and cannot be created experimentally. Instead, Bessel-like beams having a finite axial extension can be created using a phase generator such as a spatial light modulator (SLM), as described in the Methods section. This approach results in an intensity distribution near the optical axis that scales as^20^,^21^:
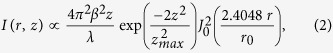
where 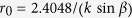 and 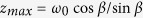 correspond to the lateral and axial full-width-at-half-maximum (FWHM) of the intensity distribution. Here the lateral and axial extensions *r*_0_ and *z*_*max*_ can be controlled independently by two experimental parameters, namely the waist of the incident Gaussian beam, *ω*_0_, and the slope of the phase modulator that determines *β*.

The combination of a beam expander and a SLM thus permits to program independently the lateral and axial extension of the focused Bessel beams through the control of the incident beam size illuminating the SLM and the propagation angle of the cone of light refracted by the SLM. As such, using a phase SLM is particularly interesting for implementing Bessel beams as it paves the way to programmable Bessel beam excitation for multiphoton microscopy.

**Figure 1:**
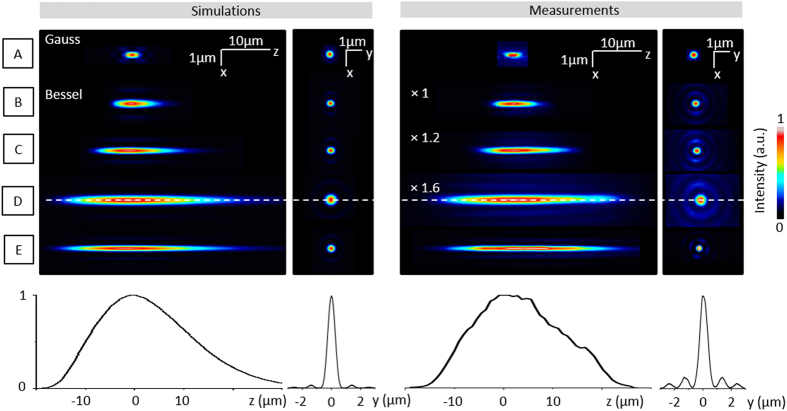
Programmable Bessel beam excitation. The figure shows the axial and transverse square intensity distribution of beams used in this study (left, simulations; right, camera-based measurements). The wavelengths and FWHM extension of the main lobe of the focused beam correspond to, from top to bottom: (**A**) (Gaussian) λ = 1100 nm, 1.4 μm × 0.3 μm; (**B**) λ = 1100 nm, 7 μm × 0.2 μm; (**C**) λ = 1100 nm, 14 μm × 0.3 μm; (**D**) λ = 1100 nm, 23 μm × 0.56 μm; (**E**) λ = 850 nm 23 μm × 0.2 μm. Profiles (bottom) are plotted along the white dotted line. Square intensity scaling factors are indicated for experimental Bessel beams obtained with the same objective and wavelength.

Figure 1 shows experimental point spread functions (PSF) measured in our microscope (see Supplementary figure S2) of Bessel beams with independent lateral and axial extensions. These results are in good agreement with the expected intensity distribution (Fig. 1 left) calculated as explained in the Methods and Supplementary figure S3. They illustrate the versatility of our design: the depth of field can be enhanced without compromising the lateral resolution of the point scanning microscope.

It should be noted however that depth-of-field extension generally comes at the price of an increase in background due to the Bessel beam secondary rings (see Supplementary information), and a decrease in excitation intensity in the main lobe if the total power of the beam is kept constant. This is illustrated in Fig. 1 and Supplementary figure S4: elongated PSFs exhibit a reduced peak intensity in the main lobe, resulting in a decreased nonlinear excitation efficiency. Moreover, axial extension also correlates with the increase in intensity in side lobes of the PSF (as visible on XY profiles and Supplementary figure S4) that induces a blur in the images obtained with such excitation. Although this blur is reduced in the case of nonlinear imaging, these two effects limit the maximum axial extension that can effectively be used for nonlinear imaging.

#### Second-harmonic generation imaging with Bessel beam excitation

**Figure 2:**
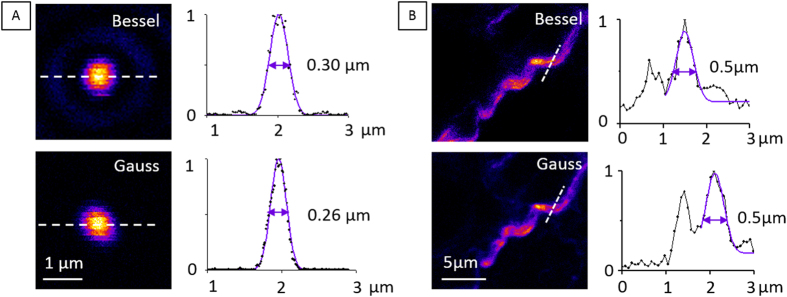
Lateral extension of the SHG images of small objects images with Bessel and Gaussian excitation. 150 nm KTP nanocrystal (**A**) and collagen fiber (**B**) imaged with Bessel (top) and Gaussian (bottom) excitation. Bessel signal was collected off-axis. Bessel beam dimensions: 7 μm × 0.3 μm. Excitation wavelength: 1100 nm. Pixel dwell times: (**A**, Bessel) 20 μs; (**A**, Gauss) 9 μs; (**B**, Bessel) 25 μs; (**B**, Gauss) 50 μs.

Figure 2 shows SHG images of the same objects recorded with Bessel and Gaussian excitation and their corresponding intensity profiles. The axial and lateral full-width-at-half-maximum of the main excitation lobe as estimated using Gaussian fits of imaging data were 7 μm × 0.3 μm for Bessel excitation and 0.7 μm × 0.26 μm for Gaussian excitation. These data confirm that the lateral resolution in the case of sparse samples corresponds to the extent of the main lobe in the case of Bessel excitation – which is consistent with results previously reported in the case of 2PEF imaging with Bessel beams^13^.

**Figure 3:**
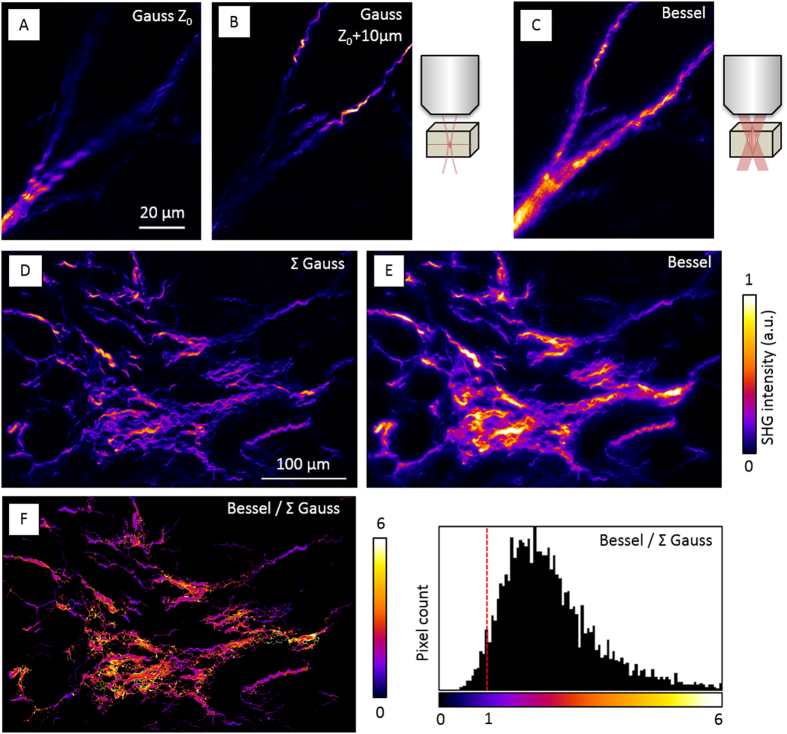
SHG microscopy with axially extended depth of field. (**A–C**) Images of collagen fibers within a 15 μm thick histological section of mouse kidney were recorded with Gaussian (0.7 μm × 0.3 μm) and Bessel (7 μm × 0.3 μm) excitations (800 nm). (**A,B**) Gaussian 2D images recorded at different depths. (**C**) 2D image of the same field recorded with Bessel excitation. (**D–F**) Comparison of SHG imaging contrast using Bessel and Gaussian excitations. (**D**) image of collagen fibers with 3D-scanned Gaussian excitation (0.7 μm × 0.3 μm) and sum projection of a Z-series. (**E**), corresponding field recorded with 2D-scanned Bessel excitation (23 μm × 0.3 μm) and off-axis detection. (**F**) Ratio of Bessel-to-Gaussian images calculated assuming similar signals for the smallest fibers. The corresponding histogram is shown on the right. Values superior to 1 reflect the coherent enhancement obtained with extended-depth Bessel excitation. Pixel dwell times, excitation powers after the objective, and maximum signal levels: (**A,B**) 65 μs, 43 mW, 250 photons; (**C**) 330 μs, 35 mW, 70 photons; (**D**) 5 μs *20, 12 mW, 250 photons/plane; (**E**) 20 μs, 45 mW, 230 photons.

Let us now analyze the ability of Bessel beams to deliver extended depth of field coherent nonlinear imaging. Figure 3A–C shows extended depth of field SHG imaging of a 15 μm-thick mouse kidney biopsy sample. Images were recorded with a 60 × 1.2 NA water immersion objective (Olympus) and at 800 nm excitation wavelength. Only off-axis SHG signal was collected in the case of Bessel excitation, for reasons discussed in the last section of this article. Collagen fibrils present in the imaged volume can be detected in a single Bessel image, while a Z-series of 15 images spaced by 0.5 μm is needed when using Gaussian excitation with similar lateral resolution. The pixel integration time used here was 3 times longer in the case of Bessel excitation, so that the imaging process was 5 times faster. Overall, these data illustrate that Bessel beams can be used to rapidly probe the SHG response of a thick sample, at the cost of losing axial resolution. 

Importantly for applications to fibrosis scoring, the signal detected during Bessel SHG imaging results from constructive interference of harmonic light scattered over the entire axial extent of the probe beam. This implies that the signal is effectively proportional to the square number of aligned harmonophores within the probe beam. This is essentially different from the case of probing a thick sample with Gaussian beams focused at successive depths, and then performing an incoherent summation of the images. In this case, the resulting contrast depends on axial sampling. Hence, Bessel beam excitation provides a different contrast mechanism. When probing a single fibril or several fibrils with the same polarity, the Bessel SHG signal is expected to scale quadratically with the density of collagen molecules. In practice, we observed in kidney histological sections that Bessel SHG highlights the most concentrated (or fibrotic) regions in the sample. This is exemplified in Fig. 3D–F showing the SHG contrast difference between Gaussian and Bessel excitation. Bessel and summed Gauss images were normalized in order to have equivalent levels from structures producing the smallest signals in both images. The ratio of Bessel-to-Gaussian signals and the corresponding pixel histogram are displayed in Fig. 3F, and illustrate how regions with thick fibrillar structures are highlighted in the Bessel image. We point out however that the quadratic dependence will be lost in samples where collagen fibril polarity is heterogeneous at the submicron scale such as cartilage, as recently analyzed by interferometric SHG (I-SHG) studies^22^. In such organizations, it remains very complex to relate SHG intensity to collagen density. Nevertheless, as suggested by Fig. 3F, extended-depth excitation does highlight fibrotic regions in kidney biopsy sections, presumably because fibrils exhibit constant polarity over thicknesses reaching several microns in this type of tissue.

#### Application to extended depth of field imaging of non-flat biopsy sections

**Figure 4:**
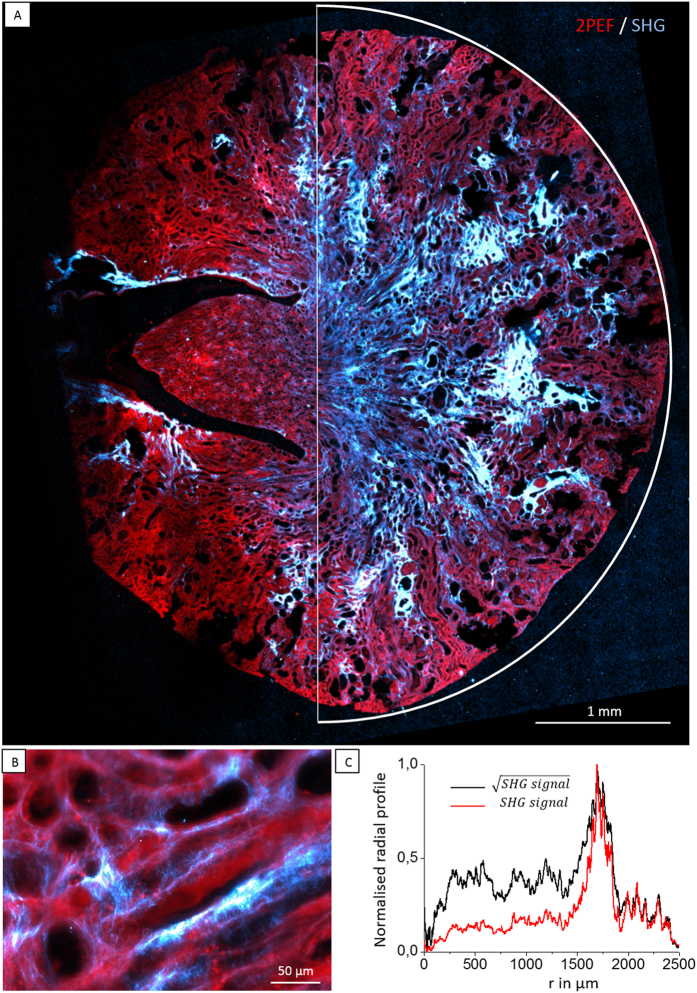
Extended-depth SHG-2PEF imaging of a fibrotic kidney histological section (see also Supplementary movie). (**A**) 15 μm thick histological section of fibrotic mouse kidney was imaged with Bessel excitation (800 nm, 23 μm × 0.2 μm) and XY mosaicking. (**A**) Stitched 2D Bessel image of the entire histological section. SHG signal is shown in blue and endogenous 2PEF is shown in red. Imaged area: 5 × 5.5 μm^2^. Lateral pixel size 0.5 × 0.5 μm^2^. Pixel dwell time, 30 μs. Power after objective, 45 mW. Signal level up to 250 photons/pixel. Scale bar, 1 mm. (**B**) Zoomed-in area showing the local SHG and 2PEF contrasts. Scale bar, 50 μm. (**C**) Integrated radial profiles of the SHG signal (red) and of the square root of the SHG signal (black).

We now demonstrate the potential of nonlinear imaging with Bessel beams for rapid probing of fibrotic collagen accumulation in thick histological sections. Using 23 μm × 0.3 μm Bessel excitation, we recorded simultaneous SHG-2PEF images of an entire section of fibrotic mouse kidney (Fig. 4 and supplementary movie). SHG was detected in the forward direction with a spatial filter selecting off-axis emission (see next section), and 2PEF was backward-detected.

The entire image spans approximately 5 × 5.5 mm^2^ and was assembled from 18 × 19 tiles recorded with 40% surface overlap for simplifying subsequent stitching, and 0.5 μm lateral pixel size. In order to detect both small and large signals without saturation, we used a pixel dwell time of 30 μs. Overall, the total exposure time was here 1 h 17 min for 154 000 000 pixels. We note that this acquisition time could be significantly reduced by using a smaller pixel dwell time (e.g. when detecting only large signals) and less overlap. Recording a Gaussian excitation image with similar lateral resolution and depth of field would have typically taken 10 times longer. The fluorescence image (shown in red) reveals the general morphology of the kidney, while the SHG image highlights fibrillar collagen.

As discussed in previous paragraphs, this imaging strategy provides data that are simpler to relate to collagen density than when using a 3D scanning scheme, since the Bessel SHG signal is proportional to the square number of in-plane collagen molecules. Note however that the collagen density may be underestimated because of partially destructive interference when collagen fibrils with anti-parallel orientations lie together within the extended focal volume^22^. A tentative map of the fibrillar collagen content in the sample can be obtained by taking the square root of the SHG image, as illustrated on the black radial profile in Fig. 4C. Alternatively, extracting a profile of the SHG signal (instead of its square root) as shown as a red profile in Fig. 4C highlights the fibrotic areas. This kidney SHG radial profile reveals that fibrosis develops around the arcuate arteries in the deep cortex of the kidney, in agreement with previous studies^5^,^23^, as well as in the renal medulla in the fibrosis model considered here (see Methods). These data illustrate that fibrosis scores can be derived from Bessel SHG images.

As explained in the introduction, the application of SHG microscopy for collagen scoring in fibrotic tissue samples requires to record extended-depth volumes because of the non-flatness of tissue biopsy samples and of the heterogeneity of collagen accumulation in fibroses. Our acquisition scheme has therefore practical advantages over the Gaussian-based approach. The acquisition of mosaic such as the one shown in Fig. 4 is faster for a given probed volume and lateral resolution. It is therefore easier with Bessel excitation to maintain mechanical stability and proper objective immersion during the imaging process. In turn, mosaic high resolution stitching can be successfully automated using standard algorithms^24^.

Overall, these data show that Bessel excitation enables an important step towards the development of systematic SHG-based diagnostics. In the next section, we analyze more precisely the physics of Bessel SHG microscopy signals.

### Properties of second-harmonic generation imaging with Bessel beam excitation

#### Signal variation with extended beam for coherent and incoherent processes

In order to investigate the physics of SHG with Bessel excitation, we consider in this section 2PEF and SHG signal levels as a function of Bessel beam axial extension, in the case of point-like and extended objects. We first discuss simple scaling arguments and corroborate them with experiments, and in the subsequent section of this article we will discuss more precisely SHG phase matching with Bessel beams.

As pointed out earlier, the energy carried by the main lobe of the Bessel beam decreases in proportion to its axial extension: in the case of a Bessel beam^25^,^26^, when the central lobe is extended axially by a factor *ρ*_*z*_ while keeping constant its lateral extension and the average laser power, the energy in the central lobe varies as 1/*ρ*_*z*_. Therefore, the signal from a point-like object is expected to decrease as 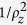 in the case of a two-photon process.

In the case of an axially extended object, the effect of beam shaping on signal level depends on whether an incoherent (e.g. fluorescence) or coherent (e.g. harmonic generation) contrast mechanism is used. In the case of 2PEF if we make the simplifying assumption that only the main lobe contributes to the excitation, the total signal is proportional to the illuminated volume times the excitation intensity squared and should scale as 1/*ρ*_*z*_ (=*ρ*_*z*_ × 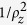). Interestingly, in the case of SHG, if all contributions scattered by the sample along the optical axis interfere in a constructive manner, the signal should scale as *ρ*_*z*_ × 1/*ρ*_*z*_^2^, i.e. should not exhibit the same drop as 2PEF from an extended sample (Table 1).

**Figure 5:**
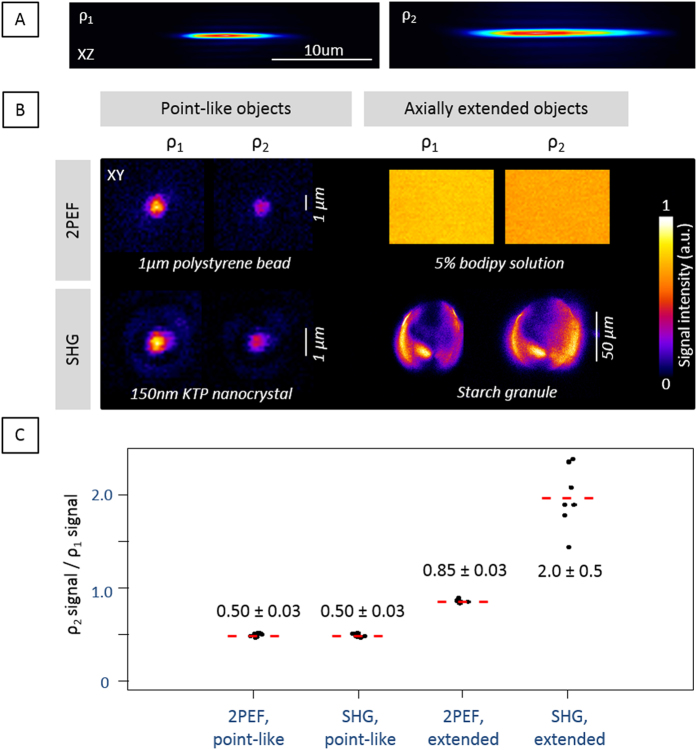
2PEF and SHG signal level for point-like and axially extended objects as a function of Bessel beam extension ρ. (**A**) Excitation PSF^2^ (square intensity distribution) estimated from camera-based measurements. Bessel beams parameters: 1100 nm, NA_B_ = 1.05, 7 μm × 0.3 μm and 14 μm × 0.3 μm extensions. (**B**) Images of point-like (150 nm KTP crystal and 1 μm fluorescent polystyrene beads) and axially extended objects (Starch granule and 5% bodipy solution) recorded with the two beams. Pixel dwell times: bead, 20 μs; KTP, 10 μs; bodipy, 5 μs; starch, 50 μs. (**C**) Ratio between the signals obtained with the two beams for 2PEF and SHG, and for each type of sample.

To experimentally validate these predictions, we recorded SHG and 2PEF signals from point-like and extended objects using Bessel beam excitation (Fig. 5). We used two shapes of Bessel beams exhibiting comparable lateral extension and different axial extensions ρ_1_ and ρ_2_ (Fig. 5A). We characterized the intensity distribution of the two excitation Bessel beams using the setup described in Supplementary figure S2: we measured the ratio between square intensities in the main lobes in the plane of focus (*z* = 0) to be 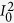
*(ρ*_*2*_*)/*
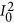
*(ρ*_*1*_) = 0.50 ± 0.05, and the ratio between integrated intensities at *z* = 0 taking into account the first 10 secondary rings to be 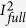
*(ρ*_*2*_*)/*
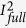
*(ρ*_*1*_) = 0.7 ± 0.1.

As a model for point-like objects, we chose samples smaller than the axial extent of our excitation beams, namely 1 μm diameter fluorescently-labelled polystyrene beads for 2PEF and 150 nm diameter KTP nanoparticles for SHG. As models for axially extended 2PEF and SHG samples, we recorded signal from a liquid bodipy solution sealed between two glass coverslips and from potato starch granules, respectively.

As summarized in Fig. 5, we found that the signal ratio *S(ρ*_*2*_*)/S(ρ*_*1*_) was 0.50 ± 0.03 in the case of small objects for both 2PEF and SHG cases. These measurements are consistent with the measured squared intensities of the excitation beams and the predictions given in Table 1, and confirm that depth-of-field extension has an important cost on 2PEF and SHG signal level from small objects. In contrast, we measured a ratio of 0.85 ± 0.03 in the case of an extended fluorescent sample, and of 2.0 ± 0.5 in the case of an axially extended harmonic generator (*i.e.* the SHG signal in this case increased with beam extension). These observations confirm that, in contrast with fluorescence, harmonic generation with Bessel beams occurs coherently over the extent of the beam, so that large SHG signals may be observed from large objects despite reduced excitation intensity. These results suggest that the extended depth of field properties of Bessel beams may be used for rapidly probing nonlinear properties of thick samples when using coherent signals. In the next section, we will analyze more specifically the signal generation mechanisms for SHG imaging.

#### Phase matching and directionality

**Figure 6:**
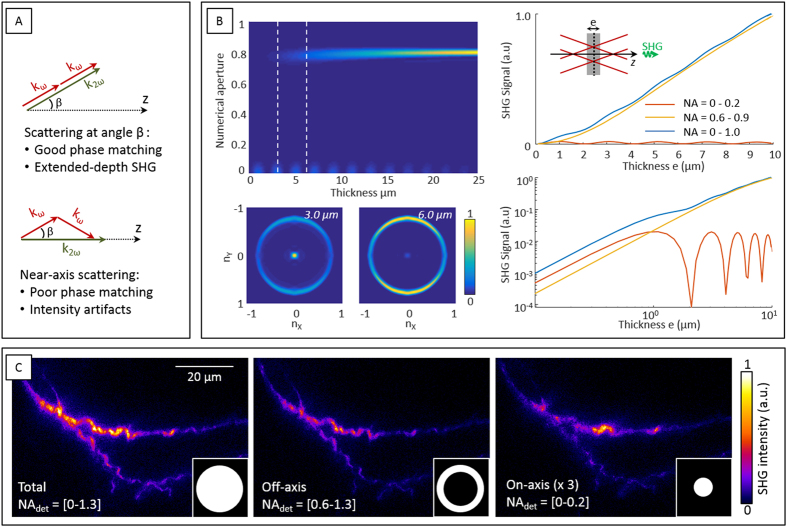
Scattering directions of SHG with Bessel beam excitation. (**A**) Phase-matching as a function of SHG scattering direction. (**B**) Top left, numerical simulations of SHG from a slab sample centered at focus as a function of sample thickness and emission direction. Off-axis emission increases with sample thickness in this range owing to good phase matching. Near-axis scattering efficiency oscillates with sample thickness. Bottom right, far-field emission diagrams for sample thicknesses of 3 μm and 6 μm. Right, integrated SHG as a function of sample thickness shown in linear and logarithmic scales, for different ranges of detection NAs: near-axis scattering (red), off-axis scattering (yellow), and total scattering (blue). Off-axis SHG scales as the square of sample thickness up to approximately 10 μm. Simulation conditions: λ = 1100 nm, NA_B_ = 0.8 ± 0.1, n = 1.33, no dispersion. (**C**) SHG images of collagen fibers recorded with Bessel excitation (800 nm, NA_B_ = 1) and angular filtering in the detection path. Signal was successively recorded with the following conditions (from left to right): detection in all angular directions with NA_det_ ranging from 0 to 1.2; detection of off-axis SHG with NA_det_ ranging from 0.6 to 1.2; detection of near-axis SHG with NA_det_ ranging from 0 to 0.2 and tripled acquisition time.

Because SHG is coherent, its efficiency and directionality depend both on sample structure and on excitation field distribution. SH radiation efficiency in a particular direction is governed by wave vector mismatch in that direction. The wave vector mismatch Δ***k***_*θ*_ between the driving and harmonic fields in a direction *θ* is given by the geometrical projection of ***k***(*2ω*) − 2***k***(*ω*) along that direction. If we consider the case of a non-dispersive medium for simplicity, with Bessel excitation characterized by an angle *β* (Supplementary figure S1), efficient second-harmonic emission occurs along direction *β*, for which wave mismatch is minimal *i.e.* coherence length is maximal. Alternatively, on-axis emission is characterized by a coherence length *Lc* = π/Δ***k***_*0*_ ≈ π/(2*k*(1 − cos*β*)) (see the schematics in Fig. 6).

To provide a more complete picture of the emission directionality, we performed numerical simulations of SHG emission directions by a sample of different thicknesses illuminated by a Bessel beam. The simulations followed the approach described in^27^ for the case of third-harmonic generation. A vector field model was used for the excitation field focused in representative conditions (λ = 1100 nm, NA_Bessel_ = 0.8 ± 0.1, n = 1.33, no dispersion). A model sample geometry was considered, corresponding to the simple case of a semi-infinite slab with configurable thickness transverse to the propagation axis. The sample was considered to be a monocrystal characterized by a unique tensor element 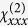 with *x* the polarization direction of the incident beam. Figure 6B shows the calculated emission directions as a function of sample thickness. The simulations corroborate the considerations on phase matching directions. For sample thicknesses up to 7–10 μm in these conditions, off-axis SHG exhibits a quadratic dependence on sample thickness, whereas on-axis SHG exhibits an oscillating behavior. The detection of off-axis SHG is therefore expected to provide an extended-depth image resulting from the coherent superposition of the contributions of all the dipoles excited in the probe volume. Alternatively, the detection of on-axis SHG provides an image characterized by a coherence length shorter than the axial extent of the Bessel beam, i.e. containing intensity artifacts related to the local thickness and axial structure of the probed sample^27^,^28^,^29^,^30^.

We experimentally validated these predictions by recording transmission SHG images with spatially filtered emission directions (Fig. 6C). We recorded images of the same samples either without filtering, or by blocking the near-axis emission with a mask, or by blocking the off-axis emission with a diaphragm. The emission spatial filter was calibrated by measuring the transmission of Bessel beams of known numerical aperture (NA). We then recorded SHG images of fibrillar collagen in a 15-μm thick histological section of fibrotic mouse kidney. The results are displayed in Fig. 6, and the following observations can be made: (i) Bessel SHG is predominantly scattered off-axis; (ii) detection of on-axis or off-axis SHG results in images exhibiting different contrast. The on-axis image exhibits some “hot spots” not present in the off-axis image. Following our analysis, the interpretation of these data is that the off-axis image can be viewed as the actual extended depth of field SHG image, while the near-axis image contains intensity variations related to the interplay between the axial spatial frequencies of the sample and the on-axis coherence length associated with the Bessel beam.

## Conclusion

Bessel beam excitation is an effective approach for increasing the depth-of-field while maintaining lateral resolution in point scanning microscopy. In this work we have analyzed and demonstrated the use of Bessel beam excitation specifically for SHG imaging, *i.e.* a coherent nonlinear process. Considering collagen imaging, we have shown that strong Bessel SHG signals are observed in dense collagen regions owing to coherence over the axially extended probe volume, despite the reduction in excitation intensity involved by beam shaping. When combined with spatially filtered detection and 2D scanning, this technique is an effective means for probing collagen accumulation in histological sections. This approach has two advantages compared to the usual imaging scheme relying on 3D scanning of a tightly focused Gaussian beam.

Firstly, the Bessel SHG signal is proportional to the square number of collagen molecules with parallel orientation within the focal plane assuming constant molecular polarity within the excitation volume, which seems to be verified for fibrotic tissues. Signals therefore have a simpler relation to collagen density than when using a 3D scanning scheme. A meaningful map of the fibrillar collagen content in fibrotic samples can be deduced from the Bessel SHG image, opening the way to fibrosis scoring. In addition, our analysis of the contrast mechanisms involved in Bessel SHG microscopy shows how spatial filtering of the detection should be used to select the signal corresponding to coherent extended depth of field emission. We note however that the quadratic relation between SHG and collagen density is lost in samples where collagen fibril polarity is heterogeneous at the submicron scale^22^. In such organizations, it remains very complex to relate SHG intensity to molecular density. Although this is beyond the scope of the present study, we note that for that purpose, Bessel excitation with adjustable depth-of-field can provide a novel contrast mechanism for analyzing collagen macromolecular organization, complementary to I-SHG and polarization-resolved SHG.

Secondly, as also illustrated in this work, the acquisition time needed to probe a histological section can be significantly reduced with Bessel excitation. An extended depth of field is needed to probe histological sections over several mm^2^ due to their poor flatness. Faster imaging and 2D scanning are advantageous as they mitigate issues associated with mechanical stability and 3D image stitching. We also note that the Bessel acquisition scheme could be further optimized for fast scanning of histological samples by using a more efficient phase modulator such as a static diffractive phase mask^13^, a laser source with higher peak power, and multipoint excitation.

Bessel excitation appears to be well suited for rapidly probing non-flat thin samples such as most tissue biopsy samples while preserving lateral resolution. Overall, this study paves the way to the development of systematic SHG-based assays for large-scale physiopathological studies.

## Methods

### Multiphoton microscopy with programmable Bessel beam excitation

Imaging was performed on a lab-built multiphoton point-scanning microscope constructed around an inverted frame (Olympus IX-70). The output beam from a Titanium-sapphire oscillator (690–1090 nm, 80 MHz, 150 fs, Chameleon Ultra 2, Coherent, USA) or an optical parametric oscillator (1000–1400 nm, 80 MHz, 350 fs, compact OPO, Coherent, USA) was sent though a variable beam expander (S6EXZ5076/328, Sill Optics) and then shaped into an annular intensity distribution using a reflective liquid crystal spatial light modulator (LCoS-SLM X10468, Hamamatsu, Japan) and a lens (Supplementary figure S2). The zeroth diffraction order was rejected after a focusing lens by incorporating a tilt on the SLM pattern and a blocking mask in the Fourier plane (see^31^ ). The transmission of the shaping module was approximately 72% at 1100 nm and 60% at 840 nm. The shaped beam was angularly scanned at the back pupil plane of the microscope objective by two conjugated galvanometric mirrors (VM500+, GSI, USA). This configuration produces an annular intensity distribution of light on the scanning mirrors and at the back pupil of the excitation objective, and a Bessel beam at its focus. Two water immersion objectives were used to focus the excitation beams in the sample: a 25 × 1.05 NA XLPlanN and a 60 × 1.2 NA UPlanSApo. Excitation intensity was controlled with a wave plate and a polarizer. Nonlinear signals were separated from the excitation light with appropriate filters (Semrock 400/20 for SHG and 475/64 for 2PEF), and detected in the epi- or trans- directions by photomultiplier tubes (SensTech, UK) and lab-designed photon counting electronics. Scanning and acquisition were synchronized using a custom LabVIEW program and a multichannel I/O board (PCI-6115, National Instruments, USA).

To measure the excitation point spread functions (PSF), we added a characterization module^29^,^30^ consisting of a removable 50/50 beam splitter, a tube lens (F = 300 mm) and a CCD camera (AVT Stingray F-125B). 3D PSFs were measured by scanning axially a silver-coated mirror across the focal plane.

We note that implementing a telecentric arrangement for the scanning system, i.e. ensuring that the two scanning mirrors are conjugated with the objective back aperture, is important to minimize aberrations and PSF tilting during the scan. With the 25× objective, this configuration resulted in a usable field of view larger than 400 μm where Bessel excitation was properly shaped and parallel to the optical axis (Supplementary figure S2).

We also note that, as in the case of Gaussian excitation, the accuracy of collagen quantification may be affected by the orientation of collagen fibrils within the excitation volume, and that this effect can be mitigated by using circularly polarized excitation.

For spatially filtering the SHG signal in wave vector space, we used the collecting condenser diaphragm or a blocking mask located just after the condenser. We note that this spatial filtering should be implemented in Fourier space, so that this configuration for the blocking mask is slightly suboptimal. In practice, we found that an acceptable trade-off between filtering and lateral field-of-view is typically obtained when blocking the smallest emission angles over two-thirds of the excitation NA.

### Bessel beam generation using a LCoS reflective spatial light modulator

The SLM used in this study could generate maximal phase shift slightly larger than 2π radians. Larger shifts were obtained through phase aliasing between 0 and 2π. As described in previous studies^32^ , the use of a SLM instead of an axicon to produce Bessel beams introduce unwanted spatial replicas and diffraction orders. We applied a phase ramp to the SLM hologram and used spatial filtering to select the first diffraction order^32^. The phase applied by the SLM was therefore of the form:
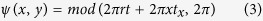
with *t* the amplitude of the conical phase and *t*_*x*_ the amplitude of the additional linear phase along the *x* direction.

In terms of light efficiency, the reflectivity of the LCoS-SLM used here is 60% (resp. 70%) at 850 nm (resp. 1100 nm), and the overall efficiency of our beam shaping system is 23% (resp. 25%) at 850 nm (resp. 1100 nm).

### Numerical simulations of focused Bessel beams generated by an SLM

To calculate field distributions at the focus of a high *NA* objective 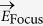, we used a reformulation of the Debye integral based on Fourier transform (FT), as proposed by Leutenegger *et al*.^33^:
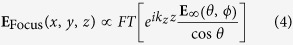
where 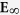 is the projection on a sphere of the incoming field 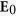 in the back aperture of the microscope objective. See also Supplementary figure S3. Thus for a field linearly polarized along *x*:
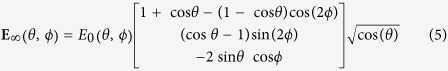


The field in the back aperture plane of the microscope objective is related to the field in the SLM plane by a Fourier transform. Therefore, if the SLM is illuminated by a Gaussian beam linearly polarized along *x*, *E*_0_ can be written as:
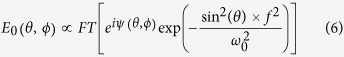
where *ω*_0_ is the waist of the Gaussian beam illuminating the SLM and *f* the focal length of the projecting lens.

### Numerical simulations of SHG signals generated by focused Bessel beams

To simulate SHG using Bessel beams, we used a simplified model for Bessel beams that is easier to relate to excitation numerical aperture. Following the approach described in^27^ for the case of third-harmonic generation, we considered an annular intensity distribution with constant radial phase focused by a high numerical aperture (NA) objective. In this case, the field in the back aperture reads:
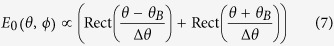
where *θ*_*B*_ describes the Bessel beam focusing angle and Δ*θ* its angular spread. These parameters are linked to *NA*_*B*_ and Δ*NA*_*B*_ by the following relations:
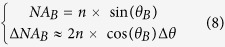


The excitation field near focus was discretized on a 201 × 201 × 251 grid with voxel size of 35 × 35 × 70 nm^3^. The nonlinear polarization induced near focus was calculated as a function of the sample geometry assuming a simplified second-order tensorial response:
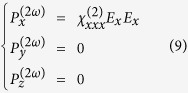


The harmonic field originating from all positions **r** in the focal region and propagated to a position 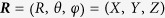 can then be expressed as^34^,^35^,^36^:
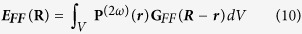
where *V* spans the excitation volume and 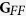 is the far field Green’s function:
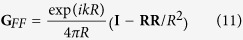
with **I** the identity matrix.

Emission diagrams were calculated over a sphere of radius R and the projection of the diagrams were displayed on a 2D plane whose axes are given by dimensionless variables 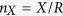 and 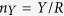.

Finally, the total radiated SHG power was calculated by integrating the far field intensity on the sphere of radius *R* with the cone angle of the objective lens:
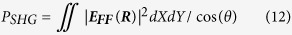


### Preparation of histological sections

Renal fibrosis was induced in wild-type mice using the AGBM model (anti-glomerular basement membrane glomerulonephritis)^37^. Mice were sacrificed after 21 days, kidney were removed and fixed and paraffin-embedded unstained coronal kidney sections deposited on glass slides were obtained. The thickness of these histological sections was chosen as 15 μm, which is slightly larger than usual histological sections (2–7 μm thick), in order to better illustrate depth of field issues when imaging large regions of sections deposited on slides with poor flatness.

All experimental animal protocols were performed in accordance with guidelines of the European Community (L358–86/609EEC), and were approved by the Institut National de la Santé et de la Recherche Médicale and local Ethic Review Board at Paris Descartes University (Paris, France).

### KTP nanoparticles

KTP (KTiOPO4) nanoparticles were produced as a colloidal suspension following the process previously described in^38^. The basic principle relies on a coprecipitation of precursors in solution followed by a thermal treatment at 700 °C of the recovered precipitate. After washing the obtained powder to remove excess KCL salt, KTP nanoparticles spontaneously disperse in water owing to a high surface charge from ionized phosphate groups (zeta potential of −45 mV in neutral water). Structural characterization show that the particles are highly crystalline, with a size distribution peaking at about 150 nm as determined from dynamic light scattering experiments.

## Additional Information

**How to cite this article**: Vuillemin, N. *et al*. Efficient second-harmonic imaging of collagen in histological slides using Bessel beam excitation. *Sci. Rep.* **6**, 29863; doi: 10.1038/srep29863 (2016).

## Supplementary Material

Supplementary Information

Supplementary Movie

## Figures and Tables

**Table 1:** Expected signal variation as a function of Bessel beam axial extension ρ_z_ for incoherent and coherent two-photon processes.

	**Point-like object**	**Axially extended object**
2PEF	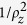	1/*ρ*_*z*_^2^ × ρ_z_ = 1/*ρ*_*z*_
SHG	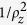	*ρ*_*z*_ × 1/*ρ*_*z*_^2^ = 1
