# Erratum: Efficient second-harmonic imaging of collagen in histological slides using Bessel beam excitation

**DOI:** 10.1038/srep31258

**Published:** 2016-08-25

**Authors:** Nelly Vuillemin, Pierre Mahou, Delphine Débarre, Thierry Gacoin, Pierre-Louis Tharaux, Marie-Claire Schanne-Klein, Willy Supatto, Emmanuel Beaurepaire

Scientific Reports 6: Article number: 29863; 10.1038/srep29863published online: 07 20 2016; updated: 08 25 2016

This Article contains typographical errors.

In the Results and Discussion section under subheading ‘Properties of second-harmonic generation imaging with Bessel beam excitation’,

“Interestingly, in the case of SHG, if all contributions scattered by the sample along the optical axis interfere in a constructive manner, the signal should scale as *ρ*_*z*_ × 1/*ρ*_*z*_^2^, i.e. should not exhibit the same drop as 2PEF from an extended sample (Table 1).”

should read:

“Interestingly, in the case of SHG, if all contributions scattered by the sample along the optical axis interfere in a constructive manner, the signal should scale as *ρ*_*z*_^*2*^ × 1/*ρ*_*z*_^2^, i.e. should not exhibit the same drop as 2PEF from an extended sample (Table 1).”

In Table 1, ‘*ρ*_*z*_^*2*^ × 1/*ρ*_*z*_^2^ = 1’ is incorrectly given as ‘*ρ*_*z*_ × 1/*ρ*_*z*_^2^’.

