# Supplementary material for: Efficient second-harmonic imaging of collagen in histological slides using Bessel beam excitation
Source: Sci Rep. 2016 Jul 20;6:29863. doi: 10.1038/srep29863 (PMC4951755; doi:10.1038/srep29863)
Supplement: Supplementary Information [file srep29863-s1.doc]

**Supplementary material**

| Article Title | Efficient second-harmonic imaging of collagen in histological slides using Bessel beam excitation |
| --- | --- |
| Authors | Nelly Vuillemin, Pierre Mahou, Delphine Débarre, Thierry Gacoin, Pierre‑Louis Tharaux, Marie‑Claire Schanne‑Klein, Willy Supatto, Emmanuel Beaurepaire |

| Supplementary Item | Title |
| --- | --- |
| Supplementary movie. | Extended-depth SHG-2PEF imaging of a fibrotic kidney histological section |
| Supplementary information. | Axial extension and lateral energy confinement in Bessel beams |
| Supplementary Figure 1 | Geometry of Bessel beam generation using a phase modulator |
| Supplementary Figure 2 | Experimental setup for Bessel beam generation and characterization in a multiphoton microscope. |
| Supplementary Figure 3 | Geometry and parameters for focus field and SHG calculations. |
| Supplementary Figure 4 | Contrast between the main lobe and secondary rings in Bessel beams. |

**Supplementary movie. Extended-depth SHG-2PEF imaging of a fibrotic kidney histological section.**

A 15µm histological section of fibrotic mouse kidney was imaged with Bessel excitation (23µm×0.2µm) and XY mosaicking. The movie illustrates various areas of the stitched 2D Bessel image of the entire histological section. SHG signal is shown in blue and endogenous 2PEF is shown in red. Imaged area: 5 ×5.5µm^2^. Lateral pixel size 0.5×0.5 µm^2^.

**Supplementary information. Axial extension and lateral energy confinement in Bessel beams.**

Axial extension and lateral energy confinement are coupled in Bessel beams, so that an increase of the depth of field results in an increase of secondary rings in transverse planes. This property can be analyzed by considering the encircled intensity as a function of the depth of field for a constant lateral extension of the main lobe:


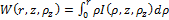
 (3),

where *I* is the intensity distribution of the focused Bessel beam and
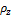
 the depth of field extension factor compared to a Gaussian beam with the same lateral extension of the central lobe. Numerical simulations indicate that the power spread of a Bessel beam increases linearly with the depth of field for a given lateral resolution, and the ratio
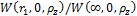
 between the intensity in the main lobe and the total integrated intensity of the beam approximately decreases as 1/ρ_z_ (see Supplementary information and Supplementary figure S4A). This phenomenon has important implications in the context of nonlinear microscopy with Bessel beam excitation. First, the excitation power must be increased by
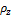
 if one wants to maintain the same intensity in the central lobe. This implies that the use of Bessel beam is costly in term of excitation power. Secondly, the background produced in a homogenously stained sample by excitation from the secondary rings also increases with
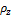
, so that extended-depth of field imaging with Bessel beams is expected to exhibit lower signal-to-background ratio (SBR), even in the case of two-photon excitation. We point out however that the situation can be different in the case of coherent nonlinear imaging such as SHG where, as we will see, emission can be spatially filtered in the far field.

Experimental issues may also reduce the contrast between the main and secondary lobes. In the example shown in Supplementary figure S4 corresponding of a Bessel beam with dimensions 23µm×0.2 µm, the contrast of the main lobe is 1/20 when integrating the excitation point spread function (PSF) in a transverse plane up to the fifth secondary ring. This contrast increases to 0.25 when considering the square of the excitation intensity, and it increases to 0.55 when considering the cube of the intensity. We attribute the reduced contrast of the main lobe compared to simulations in this particular case to the use of a large phase slope on the SLM and the bandwidth of the femtosecond laser, resulting in hologram blurring.

**Supplementary figure S1. Geometry of Bessel beam generation using a phase modulator.**

*Supplementary figure S1. Geometry of Bessel beam generation using a phase modulator. (A) A phase modulator is illuminated by an incident Gaussian beam. After propagation the phase conical modulation is converted into an intensity modulation giving rise to a Bessel-like beam. (B, C) Radial and axial intensity distribution of the corresponding beam.*

**Supplementary figure S2. Experimental setup for Bessel beam generation and characterization in a multiphoton microscope.**

*Supplementary figure S2. Experimental setup for Bessel beam generation and characterization in a multiphoton microscope. (A) Experimental setup (see text). (B) XZ projection of a 3D image of 1µm fluorescent beads, illustrating the Bessel excitation PSF across the useful field-of-view. Excitation wavelength, 1100nm, 25× objective, Bessel beam size 62µm * 0.6µm.*

**Supplementary figure S3. Geometry and parameters for focus field and SHG calculations.**


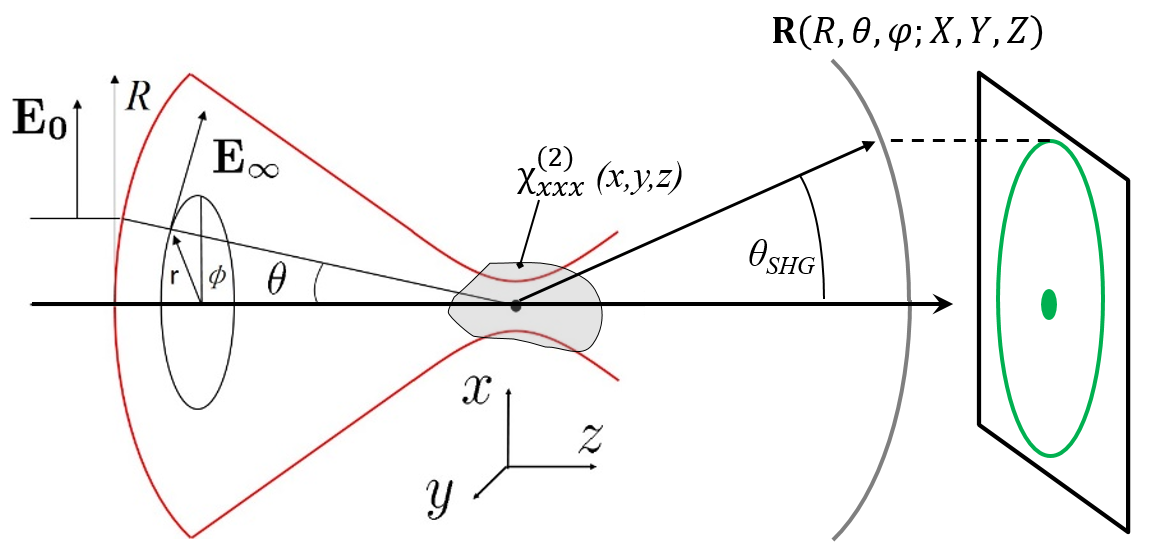


*Supplementary figure S3. Geometry and parameters for focus field and SHG calculations.*

**Supplementary figure S4. Contrast between the main lobe and secondary rings in Bessel beams.**

*Supplementary figure S4. Contrast between the main lobe and secondary rings in Bessel beams. (A) Simulation of the relative signal from the main lobe as a function of the axial extension parameter ρ_z_, for the transverse intensity distribution (PSF), its square (PSF^2^) and its cube (PSF^3^). (B, C, D) Experimental intensity profile of a Bessel beam with dimensions 23µm × 0.2µm. The figure shows XZ and XY projections of the intensity PSF, its square, and its cube. This distribution was created at λ=850nm with Bessel excitation NA=0.8.*
